# Occurrence, Ecological and Human Health Risks, and Seasonal Variations of Phenolic Compounds in Surface Water and Sediment of a Potential Polluted River Basin in China

**DOI:** 10.3390/ijerph14101140

**Published:** 2017-09-27

**Authors:** Mo Zhou, Jiquan Zhang, Caiyun Sun

**Affiliations:** 1 Institute of Natural Disaster Research, Department of Environment, Northeast Normal University, NO. 2555 Jingyue Street, Changchun 130117, China; zhoum037@nenu.edu.cn (M.Z.); xlsdydnl@126.com (C.S.); 2 Key Laboratory for Vegetation Ecology, Ministry of Education, NO. 2555 Jingyue Street, Changchun 130117, China; 3 School of Resources and Environmental Engineering, Jilin Institute of Chemical Technology, NO. 45 Chengde Street, Jilin 132022, China

**Keywords:** phenolic compounds, water, sediment, seasonal variation, ecological risks, human health risks

## Abstract

Five phenolic compounds in water and sediment of Yinma River Basin were investigated. The average concentration of phenol was the highest in water samples as well as in sediment samples during the wet season, 101.68 ng/L and 127.76 ng/g, respectively. 2,4,6-Trichlorophenol (2,4,6-TCP) and pentachlorophenol (PCP) was not detected in some sampling sites. Shitou Koumen Reservoir and the neighboring area were the severest areas of phenolic pollution. The lower reach was more polluted in three water seasons than the middle reach and upper reach. Phenol had ecological risks in sediment during three water seasons. 2-Nitrophenol (2-NP) and 2,4-dichlorophenol (2,4-DCP) had ecological risks in sediment in both the normal and wet season. The concentrations of five phenolic compounds from high to low were in the wet season, normal season, and dry season in water and sediment, respectively. There were middle risks in water of total concentrations for five phenolic compounds in several sampling sites. Total concentrations for five phenolic compounds in sediment had high ecological risks in all sampling sites. However, there was no human health risk in the Yinma River Basin.

## 1. Introduction

Phenolic compounds in the aquatic environment have drawn an extensive amount of attention from scientific institutes and environmental protection agencies due to their potential toxicity in terms of their carcinogenic, teratogenic, and mutagenetic properties which can cause adverse effects on human health [1]. Phenol, 2-nitrophenol (2-NP), 2,4-dichlorophenol (2,4-DCP), 2,4,6-trichlorophenol (2,4,6-TCP), and pentachlorophenol (PCP) are common phenolic compounds [2] which can be introduced into the environment by industrial sewage draining, agricultural irrigation, application of pesticides, and pharmaceutical drugs [3,4,5,6].

Previous studies have indicated that phenolic compounds can be detected in the environment, and can enter into aquatic organisms through the food web, and into the human body mainly through pathways of dermal contacts and ingestion [7]. As important media of the aquatic environment, water and sediment play significant roles in aquatic ecosystem [8]. There is a high likelihood that phenolic compounds in water can enter into the human body through drinking water, and the sedimentary phenolic compounds can enter into the human body via food chains [9,10]. Due to their non-biodegradable and persistent characteristics, phenolic compounds can be accumulated in the water and sediment where aquatic organisms hunt and forage; upon reaching certain levels, the exposure of phenolic compounds through water and sediment may pose threats to aquatic ecosystem and human health [11,12].

The Yinma River Basin (43°0′ N to 45°0′ N and from 124°30′ E to 126°0′ E) is located in the middle of Jilin Province of China and is surrounded by major industrial cities like Changchun, Jiutai, and Dehui. Because the basin supplies urban domestic water and agricultural irrigation water, the water quality of Yinma River is closely associated with the health of residents, and the growth condition of crops and ecosystems in the region. Due to variations in geographical locations and in the climatic zone, concentration levels of phenolic compounds in the Yinma River Basin may experience seasonal variations, which may lead to differences in exposure risk levels in three water seasons. In recent years, an increasing amount of pollution from industrial sewage draining, agricultural irrigation, the application of pesticides, and pharmaceutical drugs, all of which are regarded as potential sources of phenolic compounds, have been input into the Yinma River Basin. Nevertheless, the issue of pollution from phenolic compounds has not been paid enough attention. In the scope of our knowledge, there have hardly been any studies or reports on the pollution of phenolic compounds in the basin in recent decades. In addition, seasonal variations in hydrological conditions and environmental factors affecting the natural attenuation processes of organic pollutants in aquatic environments may lead to differences in partitioning, concentration, and risk levels [13,14]. As the Yinma River Basin experiences three distinct water seasons, seasonal variations of phenolic compounds in the aquatic environment should be clarified.

Accordingly, the aims of this study were to investigate the seasonal and spatial distribution of selected phenolic compounds in water and sediment of the Yinma River Basin, assess the exposure risks to water and sediment on ecosystem and public health, and clarify the seasonal variations.

## 2. Materials and Methods

### 2.1. Study Area, Sampling, and Sample Pre-Treatment

**Figure 1:**
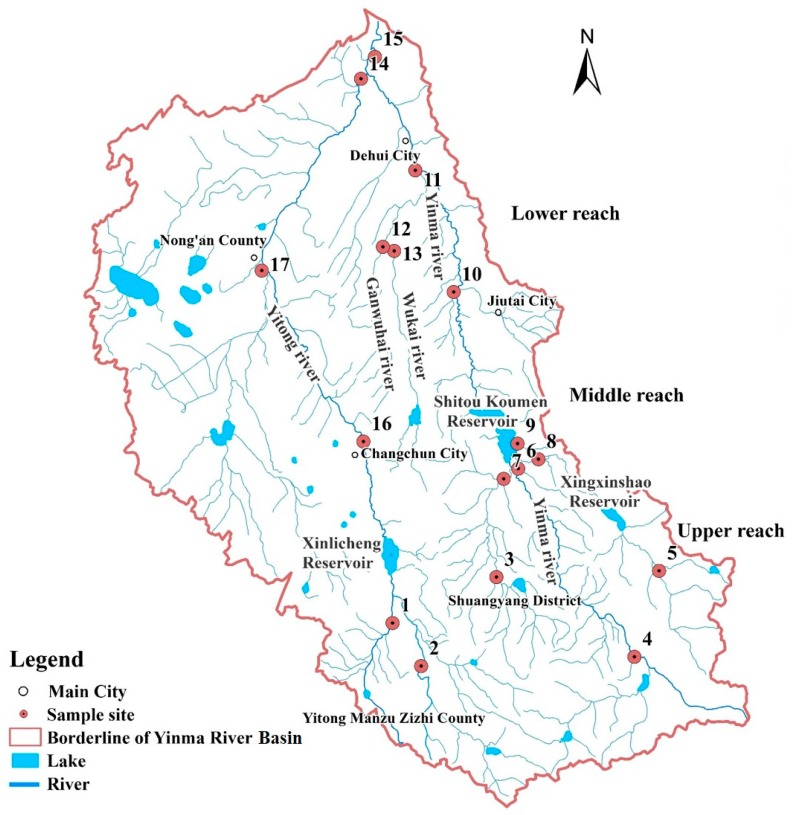
Sampling sites of the Yinma River Basin.

The Yinma River Basin (43°0′ N to 45°0′ N and from 124°30′ E to 126°0′ E) is located in the middle of Jilin Province, China, and experiences three distinct water seasons. As the Yinma River Basin is both the primary domestic and irrigation water source for the surrounding area, thirteen provincial monitoring sections and four state monitoring sections are located in the Yinma River Basin (as shown in Figure 1). To explore seasonal and spatial distribution, a total of seventeen water and sediment samples (in situ) were respectively collected from monitoring sections. Six samples were collected from Yinma River (Site 4, Site 6, Site 9, Site 10, Site 11, Site 15, with Site 4 and Site 15 located in the state monitoring sections); five samples were collected from Yitong River (Site 1, Site 2, Site 14, Site 16, Site 17, with Site 14 and Site 16 located in the state monitoring sections); two samples were collected from the flowing river of Shitou Koumen Reservoir (Site 3 and Site 5); and four samples were respectively collected from Shuangyang River (Site 7), Wukai River (Site 13), Ganwuhai River (Site 12), and Chalu River (Site 8). Sampling campaigns were performed during each of the three water seasons in May (normal season), August (wet season), and November (dry season) in 2016.

Each sample was a composite of three subsamples, with the distance between every two subsamples set to at least 200 m. Twenty liters of water (taken from 0.5–1 m below the surface) was collected for each subsample from each sampling site. The water samples were filtered with 0.45-μm glass fiber filters, and stored and labelled in brown glasses. Sediment samples were sampled with a grab sampler and freeze-dried in the dark, grounded in a mortar, sifted by a 100-mesh sieve, and stored in brown glass jars. All the samples were analyzed within 24 h.

### 2.2. Extraction Methods

#### 2.2.1. Water

One liter (1 L) of water sample that had previously been acidified to pH 2 by phosphoric acid was extracted three times with 100 mL dichloromethane (DCM) and twice with *n*-hexane (100 mL each), then the mixture of extracts was evaporated to dryness by a rotary vacuum evaporation apparatus (RE-52AA, Shanghai Yarong Inc., Shanghai, China). The final volume was reduced to 2 mL with methanol for the derivatization [15].

#### 2.2.2. Sediment

Ten grams (10 g) of each sediment sample were extracted four times in an ultrasonic bath with 50 mL of 0.1 M NaOH/methanol. The extracts were transferred through 2 L of separation funnel and the volume was reduced to 1 L with distilled water. The solution was treated with the same method as the treatment of water samples. Then the extracts were mixed with distilled water (8 mL) and passed through an Oasis HLB cartridge which was conditioned with 4 mL dichloromethane, 4 mL methanol, and 5 mL distilled water [16]. Then, 2 × 5 mL of acetonitrile were eluted from the phenolic compounds which were retained in the solid phase. Extracts were evaporated to near dryness and the final volume was increased to 2 mL with methanol for the derivatization [15].

#### 2.2.3. Derivatization of the Phenolic Compounds

Two milliliters of methanol extracts were transferred to 20-mL glass tubes with Teflon screw caps. Three milliliters (3 mL) of 0.2 M K_2_CO_3_ and 2 mL of *n*-hexane with 200 μL of acetic anhydride were mixed with the extracts, then shaken on a vortex for 2 min and left for 30 min. Then *n*-hexane layer was collected. The extraction process was repeated twice with 2 mL *n*-hexane each. Cu powder was added to remove sulfur. The final volume was reduced to 1 mL [15].

### 2.3. Quantification Methods

GCMS-QP 2010 (Shimadzu Corporation, Kyoto, Japan) was employed for the quantification of phenolic compounds. The equipment was operated in the splitless mode for 2 min at 50 mL/min in order to purge the instrument. The temperature of the injection port was maintained at 220 °C and the injection volume was 1 μL. A 30 m × 0.25 mm × 0.25 μm HP-5 capillary column was used to separate target compounds. The oven temperature was held at 40 °C for 1.5 min, increased to 100 °C for 0.5 min at 40 °C/min, increased to 120 °C at 2 °C/min, and finally increased to 300 °C at 30 °C/min and held for 5 min with a total run program of 25 min. Helium (99.999% purity) was used as a carrier gas with the column flow rate of 1 mL/min. The temperature of the transfer line, source, and quadrupole was 300 °C, 230 °C, and 150 °C, respectively. Standard electron impact condition (70 eV) was used.

### 2.4. Quality Control

All data were subjected to rigorous quality assurance. Mix standard solutions of phenol, 2-nitrophenol (2-NP), 2,4-dichlorophenol (2,4-DCP), 2,4,6-trichlorophenol (2,4,6-TCP), and pentachlorophenol (PCP) were used to perform method validation and quality control with correlation coefficients for calibration curves all higher than 0.994. Both the water samples and sediment samples were analyzed in triplicate with relative standard deviations less than 9.3%. Five parallel experiments for recovery efficiencies were checked by spiking blank samples with the mixture standard. The results indicated that recoveries for water samples were 89–120%, and for sediment samples were 68–118%. The detection limits ranged from 0.25 to 6.07 ng/L for water and from 0.53 to 9.36 ng/g for sediment.

### 2.5. Risk Assessment

#### 2.5.1. Ecological Risk Assessment

Risk quotient (RQ) was employed to calculate the potential ecological risks of each target pollutant, which is described as follows [17]:

(1) RQ=MEC/PNEC,

where MEC is the measured environmental concentration and PNEC is the predicted no effect concentration. The value of PNEC_water_ was obtained from the ratio between the acute toxicity data (Lethal Concentration 50 (LC_50_) or no observed effect concentration (NOEC) data) and an assessment factor (AF) of 1000. NOEC values were obtained from the PBT (Persistent, Bioaccumulative, and Toxic) profiler of the EPA (Environmental Protection Agency) [18]. The equilibrium partitioning method was used for calculating the PNEC_sed_ values of phenolic compounds in sediment due to the absence of toxicity data for phenolic compounds in sediment [19]; the equilibrium partitioning method is expressed as follows:

(2) $${\mathrm{PNEC}}_{\mathrm{sed}}=\frac{K_{\mathrm{susp}-\mathrm{water}}}{{\mathrm{RHO}}_{\mathrm{susp}}}\times {\mathrm{PNEC}}_{\mathrm{water}}\times 1000\times 4.6,$$

(3) $$K_{\mathrm{susp}-\mathrm{water}}=F_{\mathrm{water}-\mathrm{susp}}+F_{\mathrm{solid}-\mathrm{susp}}\times F_{\mathrm{oc}-\mathrm{susp}}\times \frac{K_{\mathrm{oc}}}{1000}{\mathrm{RHO}}_{\mathrm{solid}},$$

where RHO_susp_ is the density of wet suspended matter, which is 1150 kg/m^3^; RHO_solid_ is the density of the solid phase, which is 2500 kg/m^3^; F_water-susp_ is the volume fraction of water in suspension, which is defined as 0.9 m^3^/m^3^; F_solid-susp_ is the volume fraction of solid in suspension, which is defined as 0.1 m^3^/m^3^; F_oc-susp_ is the mass fraction of organic carbon in suspension, which is assigned as 0.1 kg/kg; K_oc_ is the partition coefficient of organic carbon-water (L/kg), which was obtained from the database of the EPI suite v4.11 software (EPI, Washington, DC, USA). The parameters are shown in Table S3.

RQ was calculated to assess the potential ecological risks by comparing the levels of phenolic compounds in water against their corresponding quality values. It is considered that RQ > 1 indicates a high risk; 0.1 < RQ < 1 indicates a medium risk; while RQ < 0.1 indicates a low risk [17].

#### 2.5.2. Human Health Risk Assessment

Health risk was characterized by non-carcinogenic risk and carcinogenic risk.

The non-carcinogenic risk is considered by the hazard quotient (HQ) as follows [20]:

(4) HQ=CDI/RfD,

(5) CDI=(C×DR×EF×ED)/(BW×AT),

(6) $${\mathrm{HQ}}_{\mathrm{total}}=\sum_{i=1}^{n}\frac{\mathrm{CDIi}}{\mathrm{RfDi}},$$

The carcinogenic risk was expressed as incremental lifetime cancer risk (ILCR) as follows:

(7) ILCR=CDI×CSF,

(8) $${\mathrm{ILCR}}_{\mathrm{total}}=\sum_{i=1}^{n}{\mathrm{CDI}}_{i}\times {\mathrm{CSF}}_{i},$$

where CDI means chronic daily intake (mg/kg·d^−1^), RfD is chronic reference dose (mg/kg·d^−1^), C is the contaminant concentration (mg/kg·d^−1^), DR is the daily consumption rate (L·d^−1^), EF is exposure frequency (d·a^−1^), ED is exposure duration (a), BW is body weight (kg), AT is averaging time (d), and CSF is cancer slope factor (kg·d/mg). RfD and CDI were collected from the IRIS (Integrated Risk Information System) database developed by the EPA, as shown in Table S3 [21].

HQ was calculated to assess the non-cancer risk by the comparison of the levels of phenolic compounds. It is considered that HQ > 1 indicates a high non-cancer risk; while HQ < 1 indicates a low non-cancer risk.

The EPA’s generally acceptable risk range for site-related exposures is 1 × 10^−4^–1 × 10^−6^. ILCR < 1 × 10^−6^ indicates that the risk is negligible, while ILCR > 1 × 10^−4^ is considered to be an unacceptable risk level [22].

## 3. Results

### 3.1. Occurrence of the Five Phenolic Compounds in Water and Sediment

The descriptive data for the concentrations of five phenolic compounds in water and sediment are presented in Table 1. The results indicated that all of the five phenolic compounds were detected in the water and sediment from the Yinma River Basin. 2-NP and 2,4-DCP were detected in all of the water and sediment samples, but 2,4,6-TCP, and PCP were not detected in some samples. The total concentrations of the five phenolic compounds in water samples varied from 84.07 ng/L to 280.04 ng/L with a mean value of 194.64 ng/L in the normal season, from 103.67 ng/L to 331.95 ng/L with a mean value of 242.6 ng/L in the wet season, and from 52.83 ng/L to 230.03 ng/L with a mean value of 153.29 ng/L in the dry season; the total concentrations of the five phenolic compounds in sediment samples varied from 149.49 ng/g to 348.60 ng/g with a mean value of 247.95 ng/g in the normal season, from 188.14 ng/g to 448.09 ng/g with a mean value of 326.83 ng/g in the wet season, and from 96.30 ng/g to 250.00 ng/g with a mean value of 173.57 ng/g in the dry season. The mean concentrations were all below PNEC_water_ and PNEC_sed_ values in the three water seasons expect phenol in all three seasons and 2-NP and 2,4-DCP in sediment in the wet season.

#### 3.1.1. Spatial-Seasonal Variations of the Five Phenolic Compounds in Water

**Figure 2:**
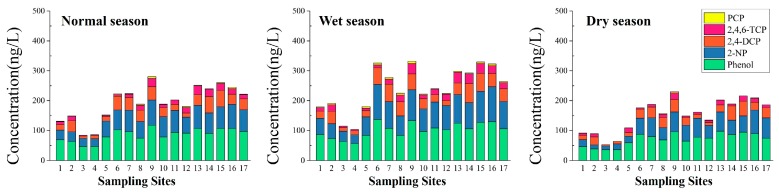
Spatial and seasonal distributions of the concentrations of five phenolic compounds in water from different sampling sites of the Yinma River Basin. PCP: pentachlorophenol; 2,4,6-TCP: 2,4,6-trichlorophenol; 2-NP: 2-nitrophenol; 2,4-DCP: 2,4-dichlorophenol.

Figure 2 illustrates spatial-seasonal variations in the concentrations of five phenolic compounds in water. Among the five phenolic compounds, the successive order of concentration in water was phenol > 2-NP > 2,4-DCP > 2,4,6-TCP > PCP.

In the normal season, the highest concentration of phenol and 2-NP (117.85 ng/L and 84.32 ng/L, respectively) were both found in the Shitou Koumen Reservoir (Site 9). Lower concentrations of phenol were observed at Site 3 and Site 4, with concentrations below 50 ng/L. As for 2-NP, Sites 1–4 we observed to have lower concentrations, with values below 35 ng/L. The highest concentration of 2,4-DCP was found in the lower reach of Yinma River (Site 15), with a value of 54.83 ng/L, followed by the lower reach of Yitong River (Site 14), with a value of 54.83 ng/L. The lowest concentrations of 2,4-DCP and 2,4,6-TCP both occurred in Site 3, which is the lower reach of Shuangyang Reservoir (Site 3). The middle reach of Ganwuhai River (Site 13) had the highest concentration of 2,4,6-TCP, with a value of 29.98 ng/L. The concentrations of PCP in the sampling sites were below 5 ng/L, except at Site 9 where it was 5.47 ng/L. PCP was not detected in Sites 3, 4, and 11. 

In the wet season, the highest concentrations of phenol and 2-NP (137.35 ng/L and 116.98 ng/L, respectively) were both found in the reservoir entrance of Shitou Koumen Reservoir of Yinma River (Site 6). The lowest concentrations of phenol and 2-NP were found in the monitoring section of Yinma River (Site 4). Sites 9, 15, and 16 displayed medium concentration levels, with values beyond 100 ng/L for 2-NP. 2,4-DCP was found in the highest concentration at Site 14, with the value of 63.78 ng/L and at the lowest concentration in Site 3 (11.45 ng/L). The highest concentration of 2,4,6-TCP was found in Site 13 with a value of 35.66 ng/L. Site 4 still displayed the lowest concentration of 2,4,6-TCP. Site 9, with a value of 1.47 ng/L, was the sampling site with the highest concentration of PCP. PCP was not detected in Sites 3 and 4. 

In the dry season, the highest concentration of phenol (with a value of 97.85 ng/L) occurred in the middle reach of Ganwuhai River (Site 13), followed by Shitou Koumen Reservoir (with the value of 95.88 ng/L). The concentrations in Sites 1–4 were below 50 ng/L. The highest concentration of 2-NP, which occurred at Site 16 (Sihua Bridge of Changchun City), was 79.24 ng/L. The concentrations in other sampling sites were all below 70 ng/L. Like the normal season, the highest and the lowest concentrations of 2,4-DCP occurred in Site 15 (with 49.38 ng/L) and Site 3 (with 4.37 ng/L), respectively. The highest concentrations of 2,4,6-TCP (with a value of 21.88 ng/L) and PCP (with a value of 3.92 ng/L) were found at Site 9. 2,4,6-TCP was not detected in Sites 3 and 4. PCP was not detected in Sites 3–5, 10, 11, 14, 15, and 17.

**Figure 3:**
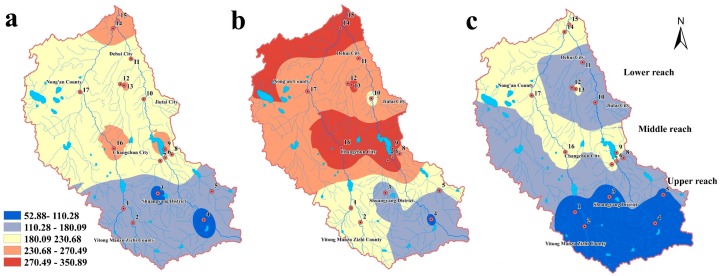
Spatial distribution of the total concentrations of the five phenolic compounds in water from the Yinma River Basin in three water seasons: (**a**) normal season, (**b**) wet season, (**c**) dry season.

Figure 3 illustrates the spatial-seasonal distribution of the total concentrations of the five phenolic compounds in water. Due to the diversity of the sources and discharges into different sampling sites, obvious spatial variations were observed.

In the normal season, the second highest level of total concentrations of the five phenolic compounds occurred at nearby regions of Shitou Koumen Reservoir, Changchun City, the lower reach of Yitong River and Yinma River, and the middle reach of Ganwuhai River. The lowest levels occurred in Shuangyang District and the middle reach of Ganwuhai River. The highest level of pollution from phenolic compounds occurred in the wet season and covered a large range of the Shitou Koumen Reservoir, Changchun City, the lower reach of Yitong River and Yinma River, and the middle reach of Ganwuhai River, all of which are located in the lower reach of the study area. Only a small portion of the monitoring section of the Yinma River displayed the lowest levels of phenolic compounds in the wet season. In the dry season, there were only intermediate levels of total concentrations in the lower reach of Yinma River Basin and the lowest levels were observed in the upper reach.

#### 3.1.2. Spatial-Seasonal Variations of the Five Phenolic Compounds in Sediment

**Figure 4:**
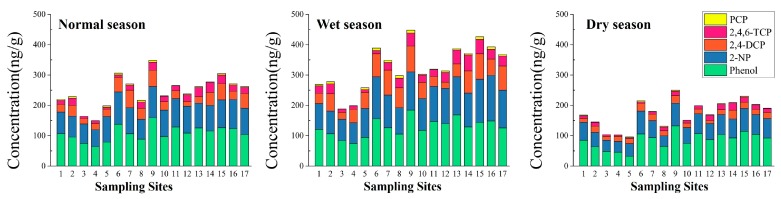
Spatial and seasonal distributions of concentrations of five phenolic compounds in sediment from different sampling sites of the Yinma River Basin.

Figure 4 illustrates spatial-seasonal variations in the concentrations of the five phenolic compounds in sediment. Among the five phenolic compounds, the successive order of the concentration in sediment was phenol > 2-NP > 2,4-DCP > 2,4,6-TCP > PCP.

In the normal season, the highest concentration of phenol was found in Shitou Koumen Reservoir (Site 9) with a value of 159.47 ng/g, followed by Site 6 with a value of 137.79 ng/g. Lower concentrations below the PNEC_sed_ value for phenol (80.35 ng/g) were observed only in Sites 3–5. The highest concentration of 2-NP, with a value of 106.63 ng/g, was found in the reservoir entrance of the Shitou Koumen Reservoir of Yinma River (Site 6), followed by Site 9, with a value of 102.52 ng/g. Concentrations beyond the PNEC_sed_ value of 2-NP (93.16 ng/g) occurred at Sites 6, 9, 11, and 16. As for 2,4-DCP, higher concentrations were found in the reservoir entrance of the Shitou Koumen Reservoir of Shuangyang River (Site 7), with 58.06 ng/g. Concentrations beyond the PNEC_sed_ value of 2,4-DCP (41.70 ng/g) were found at Sites 6, 7, 9, 14, 15, and 17. The highest concentration of 2,4,6-TCP, with a value of 32.77 ng/g (which was below the PNEC_sed_ value of 2,4,6-TCP of 61.64 ng/g) was found in the lower reach of Yitong River (Site 14), followed by the middle reach of Ganwuhai River (Site 13), with a value of 30.63 ng/g. The lowest concentration occurred in Xingxingshao Reservoir (Site 5), with 6.63 ng/g. The highest concentrations of PCP were below the PNEC_sed_ value of PCP (28.97 ng/g), with a value of 6.24 ng/g in Shitou Koumen Reservoir (Site 9). PCP was not detected in Sites 3 and 4.

In the wet season, the highest concentration of phenol, with a value of 184.63 ng/g, occurred in Shitou Koumen Reservoir (Site 9). A concentration of phenol below the PNEC_sed_ value (80.35 ng/g) only occurred in the monitoring section of Yinma River (Site 4), with a value of 74.50 ng/g. The highest concentration of 2-NP was 150.40 ng/g, which was found in Site 16. Concentrations below the PNEC_sed_ value of 2-NP (93.16 ng/g) occurred at Sites 1–4 and 8. As for 2,4-DCP, the highest concentration, with a value of 85.52 ng/g, occurred in Shitou Koumen Reservoir (Site 9). Concentrations beyond the PNEC_sed_ value of 2,4-DCP (41.70 ng/g) were found at Sites 1, 3, 4, 11, and 12. The highest and the lowest concentrations of 2,4,6-TCP occurred in the lower reach of Yitong River (Site 14), with 52.63 ng/g, and Xingxingshao Reservoir (Site 5), with 9.62 ng/g, respectively. The concentration of 2,4,6-TCP in all sampling sites were below PNEC_sed_ of 2,4,6-TCP (61.64 ng/g). The highest concentration of PCP was found in Shitou Koumen Reservoir (Site 9), with a value of 9.42 ng/g. The concentrations of PCP in all sampling sites were below the PNEC_sed_ value of PCP (28.97 ng/g).

In the dry season, for phenol, the highest concentration was still found in Shitou Koumen Reservoir (Site 9), with a value of 132.46 ng/g, and the lowest was 32.60 ng/g, which occurred in Xingxingshao Reservoir (Site 5). Concentrations of phenol below the PNEC_sed_ value of phenol (80.35 ng/g) occurred at Sites 2–5, 8, and 10. A concentration of 75.41 ng/g of 2-NP was found in the reservoir entrance of Shitou Koumen Reservoir of Yinma River (Site 6), which was below the PNEC_sed_ value of 2-NP (93.16 ng/g). The highest concentrations of 2,4-DCP and 2,4,6-TCP were both found in the lower reach of Yitong River (Site 14), with concentrations of 29.18 ng/g and 23.88 ng/g, respectively. The lowest concentration of 2,4-DCP, with a value of 10.62 ng/g, was found in the middle reach of Wukai River. The lowest concentration of 2,4,6-TCP, which was 3.32 ng/g, occurred in Xingxingshao Reservoir (Site 5). Higher concentrations of PCP were found in Shitou Koumen Reservoir (Site 9), with a value of 3.63 ng/g, and there was no PCP detected in Sites 3, 4, and 11. The concentrations of the five phenolic compounds were below each of their respective PNEC_sed_ values in the dry season, with the exception of the phenol concentrations detected at Sites 2–5, 8, and 10.

The concentrations of phenol, 2-NP, and 2,4-DCP were beyond their respective PNEC_sed_ values in the normal and wet seasons at some sampling sites. In the dry season, there was no concentration of the five contaminants beyond the PNEC_sed_ values of the five phenolic compounds at 17 sampling sites. Among them, concentrations of phenol and 2-NP were below their respective PNEC_sed_ values in only a few sampling sites. This indicated that there were risks in the sediment from phenol, 2-NP, and 2,4-DCP. This phenomenon might be because of the accumulation of phenolic compounds by sorption of sediment. 

**Figure 5:**
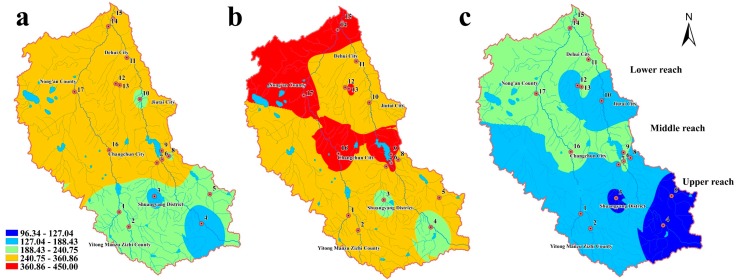
Spatial distribution of the total concentrations of the five phenolic compounds in sediment from the Yinma River Basin in three water seasons: (**a**) normal season; (**b**) wet season; (**c**) dry season.

Figure 5 illustrates the spatial-seasonal distribution of the total concentrations of the five phenolic compounds in sediment. Due to the diversity of the sources and discharges into different sampling sites, obvious spatial variations were observed. 

In the normal season, the second-highest level of total concentrations of the five phenolic compounds occurred in the lower and middle reach of the Yinma River Basin. The upper reach displayed concentrations that were of the intermediate and second lowest levels. The highest level of phenolic compounds pollution only occurred in the wet season and covered a large range of Shitou Koumen Reservoir, Changchun City, the lower reach of Yitong River and Yinma River, and the middle reach of Ganwuhai River, all of which are located in the lower reach of the study area, and Nong’an City. An intermediate level was found in Shuangyang Reservoir and the monitoring section of Yinma River. Other areas displayed the second highest level of concentration in the wet season. In the dry season, the lower reach of the study area still displayed higher concentrations, which were at the intermediate level, and the upper reach displayed the lowest levels.

### 3.2. Risk Assessment

#### 3.2.1. Ecological Risk Assessment

Table 2 indicates the risk quotient (RQ) for five phenolic compounds in water and sediment from all the sampling sites in the three water seasons. Seasonal variations in RQ for the five phenolic compounds in water were observed. The values of RQ for the three water seasons decreased in the following order: wet season > normal season > dry season. The values of RQ for the five phenolic compounds in all of the water samples were less than 0.1 in the normal and dry seasons. In the wet season, the values of RQ for 2,4,6-TCP and PCP were 0.105 and 0.126, respectively. The values of RQ for the five compounds in total were greater than 0.1. These results indicated that there was a medium level of risk in water in three water seasons and a medium risk level from 2,4,6-TCP and PCP in the wet season. As shown in Table 2, in sediment, high risks from phenol in three water seasons, and from 2-NP and 2,4-DCP in the normal and wet seasons were observed. Medium risk levels from 2,4,6-TCP and PCP were observed in the three water seasons, and there were medium risks from 2-NP and 2,4-DCP in the dry season.

**Figure 6:**
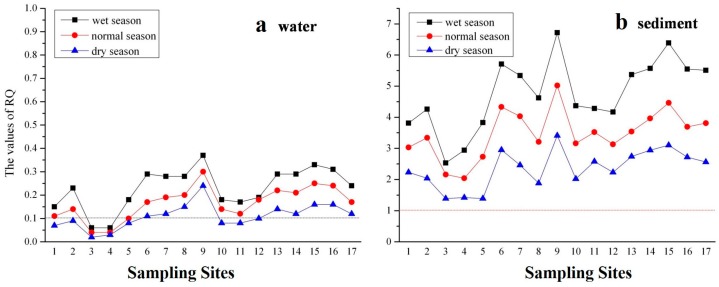
Total risk quotient (RQ) values of five phenolic compounds in 17 sampling sites in water (**a**) and sediment (**b**).

Figure 6 illustrates the total RQ values of the five phenolic compounds in 17 sampling sites in water and sediment. As shown in Figure 6a, the risks in different sampling sites were similar in the three water seasons. There were no risks at Sites 3 and 4 in three water seasons because the RQ values were less than 0.1. In the dry season, there was no risk in Sites 1–5 and 10–12. Other RQ values in water were more than 0.1 but less than 1, which means that there were medium levels of risk in water at most of sampling sites in the study area. Figure 6b illustrates that there were high risks in sediment at all three water seasons, as the values of RQ were all above 1.

#### 3.2.2. Human Health Risk Assessment

Non-carcinogenic risk and carcinogenic risk of phenolic compounds were employed to characterize the human risk assessment. Carcinogenic risk assessment is commonly based on the assumption that the tumor dose-response curve at low doses is linear and passes through the origin. Non-carcinogenic risk assessment is based on a nonlinear model. However, the carcinogenic and non-carcinogenic risk of chemicals cannot be completely distinguished [23]. In this study, in considering of the dose threshold of the chemicals, any risk that was less than the dose threshold was assumed to pose no risk for human health. 

**Figure 7:**
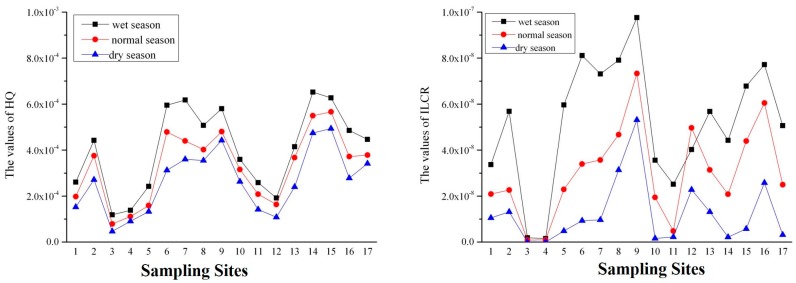
Total hazard quotient (HQ) and incremental lifetime cancer risk (ILCR) of five phenolic compounds in 17 sampling sites.

Hazard quotient (HQ) was employed to assess the non-carcinogenic risk in this study. Due to the absence of some parameters, only three phenolic compounds, phenol, 2,4-DCP, and PCP, were considered. As shown in Table S4, the values of the pollutants were less than 1 in three water seasons. Incremental lifetime cancer (ILCR) of 2,4,6-TCP and PCP is shown in Table S5. The values of ILCR were all less than 1.00 × 10^−6^. As shown in Figure 7, values of HQ were both below 1 and ILCR were below 1 × 10^−6^ at all sampling sites in the three water seasons. Higher risks were observed at Sites 2, 6–9, and 14–16.

## 4. Discussion 

### 4.1. Occurrence of the Five Phenolic Compounds in Water and Sediment

The results of the occurrence of the five phenolic compounds suggested that the pollution levels of the five contaminants were low in water, but that there was phenolic pollution in the sediment of the Yinma River Basin. However, the exposure risks of phenolic compounds from water and sediment on aquatic ecosystem and human health should be further evaluated. The values of CV (Coefficient of Variation) for some phenolic compounds in water and sediment samples were within 16–140%, indicating anthropogenic input into the aquatic environment. The concentrations of the five phenolic compounds were below each of their respective PNEC_water_ values in all three water seasons at 17 sampling sites. However, the potential risks of the five phenolic compounds need further evaluation. The order of the pollutant concentrations in water and sediment was as follows: phenol > 2-NP > 2,4-DCP > 2,4,6-TCP > PCP. The concentrations of phenolic compounds in water increase with the increase in solubility, which may be due to higher-soluble chemicals having a greater tendency of dissolving in water [24].

As indicated by the results of the spatial distribution of phenolic compounds in the Yinma River Basin, the pollutants might come from upper reach and middle reach areas with the current of the river, therefore, leading to the concentrations in the lower reach being the highest. Changchun is the capital city of Jilin Province; household garbage and industrial production are considered to be the primary sources for the phenolic pollution in aquatic environments in this area [25]. In addition, Shitou Koumen Reservoir, with elevated concentrations, receives pollution from runoffs, agriculture, and fish farming. According to Figure 3 and Figure 5, higher pollution levels occurred at Site 13, which is located near farmland areas, pesticide application may induce the pollution of phenolic compounds in aquatic environment by farmland irrigation and runoffs [26]. Due to the sorption of sediment, pollutants in sediment mostly come from the water. The results of this study indicated that sediment concentrations in the lower reach were higher than the other reaches.

The total concentration levels in water and sediment of the Yinma River Basin displayed seasonal variations, which may be due to the variations in hydrological conditions and environmental factors such as temperature and sunlight intensity. Figure 3 and Figure 5 indicated that the highest values and widest ranges of total concentrations occurred in the wet season. The lowest levels occurred in the dry season. The reason behind this phenomenon might be the increasing input of pollutants into the aquatic environment along with runoffs in water and sorption of sediment from different concentrations of pollutants.

### 4.2. Risk Assessment

The total RQ values of the five compounds in sediment indicated that there were high ecological risks in the sediment of this area. As such, additional attention should be paid to the ecological risks posed by the sediment in this area, which might have influence on farmland, stockbreeding, or cause additional pollution in water by desorption of sediment.

The highest risks in water and sediment were both located in Shitou Koumen Reservoir in the three water seasons. The RQ values at Sites 3–5 and 10–12 were lower than Sites 2, 6, 9, and 15. This might relate to the sources of phenolic compounds. Site 2 is located near the sewage drain of the sewage treatment plant where domestic and industrial sewage might accumulate. The region of Shitou Koumen Reservoir (Sites 6–9) had high risks because three main tributaries (Yinma River, Shuangyang River, and Chalu River) with various kinds of contaminants along the watercourse joined in Shitou Koumen Reservoir. Biodegradation of pesticide or pharmaceutical drugs might be the source of pollutants for Site 15 because a factory that produces veterinary medicines and farm land were in the surrounding neighborhood.

Non-carcinogen risk assessment values of phenol, 2,4-DCP, and PCP were less than 1, which means that the three pollutants had no non-carcinogenic risk in all three of the water seasons. Values of carcinogen risk assessment were less than the threshold 1.0 × 10^−6^, which means that 2,4,6-TCP and PCP presented no carcinogenic risk. The values of HQ and ILCR were lower than the threshold value. Therefore, there was no human health risk of phenolic compounds in the Yinma River Basin.

## 5. Conclusions

This work mainly investigated spatial-seasonal distribution, potential pollution sources, and seasonality in the risks of five phenolic compounds in the water and sediment of the Yinma River Basin. The results indicated that the concentrations of five phenolic compounds in water were all below the PNEC values and several concentrations of contaminants in specific water seasons were beyond the PNEC values in sediment, suggesting that phenolic pollution levels in water were low, but that there was phenolic pollution in sediment. The highest concentrations in water and sediment occurred in the lower reach of the drainage basin and the main reservoir, suggesting that the pollution of phenolic compounds accumulated along with runoffs from the input of the pollutants into the aquatic environment. The results for risk quotient indicated that there were low ecological risks from the five phenolic compounds in water. However, there were high ecological risks of the total phenolic compounds in sediment. Based on the results for hazard quotient and incremental lifetime cancer, there were low risks of phenolic compounds to human health in the Yinma River Basin.

## Figures and Tables

**Table 1:** Phenolic compound concentration ranges and mean values of 17 sampling sites for water and sediment across three water seasons in 2016. Normal Season: May; Wet Season: August; Dry Season: November.

	Water (ng/L)								
	Normal Season			Wet Season			Dry Season		
	Range	Mean	CV%	Range	Mean	CV%	Range	Mean	CV%
**Phenol**	46.75–117.85	86.33	24.3	56.73–137.35	101.68	23.9	35.72–97.85	71.04	29.9
**2-NP**	25.44–84.32	59.75	32.5	28.77–116.98	78.86	33.7	12.74–79.24	46.32	45.6
**2,4-DCP**	7.59–54.83	31.71	47.3	11.45–63.78	38.3	44.7	4.37–49.38	25.84	53.0
**2,4,6-TCP**	2.73–29.98	14.68	58.2	4.62–35.66	19.89	51.6	ND-21.88	9.31	63.9
**PCP**	ND-5.47	2.16	72.9	ND-7.34	3.86	55.7	ND-3.92	0.79	139.7
**Total**	84.07–280.04	194.64	29.9	103.67–331.95	242.6	29.9	52.83–230.03	153.29	35.6
**Phenol**	63.90–159.47	108.31	22.42	74.50–184.63	127.76	23.1	32.60–132.46	85.32	31.5
**2-NP**	56.17–106.63	83.24	16.8	67.89–150.40	108.41	23.3	34.53–75.41	56.57	24.3
**2,4-DCP**	15.75–58.06	34.22	40.7	20.52–85.52	56.46	38.0	10.63–29.18	18.48	27.3
**2,4,6-TCP**	6.63–32.77	18.95	42.8	9.62–52.63	28.74	46.7	3.32–23.88	11.53	48.3
**PCP**	ND-6.24	3.23	60.1	0.40–9.43	5.47	50.8	ND-3.65	1.66	74.0
**Total**	149.49–348.60	247.95	20.4	188.14–448.09	326.83	22.7	96.30–250.00	173.57	26.54

**Table 2:** The risk quotient values of five phenolic compounds in the Yinma River Basin.

	Water			Sediment		
RQ	Normal Season	Wet Season	Dry Season	Normal Season	Wet Season	Dry Season
**Phenol**	0.033	0.038	0.027	1.985	2.298	1.649
**2-NP**	0.030	0.042	0.028	1.145	1.614	0.809
**2,4-DCP**	0.069	0.081	0.063	1.392	2.051	0.700
**2,4,6-TCP**	0.088	0.105	0.064	0.532	0.854	0.387
**PCP**	0.094	0.126	0.068	0.215	0.325	0.126
**total**	0.315	0.392	0.250	5.269	7.142	3.671

## Supplementary Materials

The following are available online at www.mdpi.com/1660-4601/14/10/1140/s1, Table S1: The selected physicochemical properties of phenolic compounds, Table S2: The selected properties of sediment and water samples, Table S3: The selected parameters of five phenolic compounds for risk assessment, Table S4: The values of hazard quotient of five phenolic compounds in Yinma River Basin, Table S5: The values of incremental lifetime cancer (ILCR) of five phenolic compounds in Yinma River Basin.
