# Supplementary material for: Occurrence, Ecological and Human Health Risks, and Seasonal Variations of Phenolic Compounds in Surface Water and Sediment of a Potential Polluted River Basin in China
Source: Int J Environ Res Public Health. 2017 Sep 27;14(10):1140. doi: 10.3390/ijerph14101140 (PMC5664641; doi:10.3390/ijerph14101140)
Supplement: Supplementary file 1 [file ijerph-14-01140-s001.pdf]

Article

Occurrence, Ecological and Human Health Risks, and Seasonal Variations of Phenolic Compounds in Surface Water and Sediment of a Potential Polluted River Basin in China

Mo Zhou [1,2], Jiquan Zhang [1,2,*] and Caiyun Sun [1,3]

[1] Institute of Natural Disaster Research, Department of Environment, Northeast Normal University, NO. 2555 Jingyue Street, Changchun 130117, China; zhoum037@nenu.edu.cn (M.Z.); xlsdydnl@126.com (C.S.)
[2] Key Laboratory for Vegetation Ecology, Ministry of Education, NO. 2555 Jingyue Street, Changchun 130117, China
[3] School of Resources and Environmental Engineering, Jilin Institute of Chemical Technology, NO. 45 Chengde Street, Jilin 132022, China
* Correspondence: zhangjq022@nenu.edu.cn; Tel.: +86-135-9608-6467

Table S1. The selected physicochemical properties of phenolic compounds.

Chemical Name	Chemical Formula	Molecular Weight	Solubility (mg/L)	logKow
phenol	C_6H_5OH	94.11	26160	1.51
2-Nitrophenol	$C_6H_5N_1O_3$	139.11	2500	1.91
2,4-Dichlorophenol	$C_6H_4Cl_2O_1$	163	614.2	1.06
2,4,6-Trichlorophenol	$C_6H_3Cl_3O_1$	197.45	121	3.45
Pentachlorophenol	$C_6H_1Cl_5O_1$	266.34	3.09	4.74

Table S2. The selected properties of sediment and water samples.

Water	Temperature (℃)	pH	DO (mg/L)	NH_3-N (mg/L)	COD (mg/L)
Normal season	13	6.5	6.25	0.49	27.75
Wet season	25	6.5	8.1	0.56	38.85
Dry season	5	6.5	4.15	0.49	62.16
Sediment	pH	Organic matter (%)			
Normal season	7.5	3.26			
Wet season	7.02	2.84			
Dry season	7.23	2.41			

Table S3. The selected parameters of five phenolic compounds for risk assessment.

	$PNEC_{water}$ (ng·L^{-1})	RfD (mg·kg^{-1}·d^{-1})	CSF (kg·d·mg^{-1})	K_{oc} (L·kg^{-1})	$PNEC_{sed}$ (ng·g^{-1})
Phenol	3600	3.00×10^{-1}	NR	187.2	80.352
4-NP	2800	NR	NR	296.7	93.156
2,4-DCP	790	3.00×10^{-3}	NR	491.8	41.6962
2,4,6-TCP	340	NR	1.10×10^{-2}	1777	61.642
PCP	58	5.00×10^{-2}	4.00×10^{-2}	4959	28.971

NR meant there were no reports about the paramenters.

Table S4. The values of hazard quotient of five phenolic compounds in Yinma River Basin.

HQ	Normal Season	Wet Season	Dry Season
Phenol	1.16×10^{-5}	1.35×10^{-5}	9.59×10^{-6}
2-NP	--	--	--
2,4-DCP	5.38×10^{-4}	6.25×10^{-4}	4.84×10^{-4}
2,4,6-TCP	--	--	--
PCP	3.22×10^{-5}	4.31×10^{-5}	2.30×10^{-5}
total	5.81×10^{-4}	6.82×10^{-4}	5.17×10^{-4}

Table S5. The values of incremental lifetime cancer (ILCR) of five phenolic compounds in Yinma River Basin.

ILCR	Normal Season	Wet Season	Dry Season
Phenol	--	--	--
2-NP	--	--	--
2,4-DCP	--	--	--
2,4,6-TCP	9.7×10^{-9}	1.15×10^{-8}	7.08×10^{-9}
PCP	6.43×10^{-8}	8.63×10^{-8}	4.61×10^{-8}
total	7.40×10^{-8}	9.78×10^{-8}	5.31×10^{-8}
